# Determining the Presence of Superantigens in Coagulase Negative Staphylococci from Humans

**DOI:** 10.1371/journal.pone.0143341

**Published:** 2015-11-23

**Authors:** Christopher S. Stach, Bao G. Vu, Patrick M. Schlievert

**Affiliations:** Department of Microbiology, University of Iowa Carver College of Medicine, Iowa City, Iowa, United States of America; Universitätsklinikum Hamburg-Eppendorf, GERMANY

## Abstract

Superantigens (SAgs) are important virulence factors in *S*. *aureus*. Recent studies identified their presence in animal coagulase-negative staphylococci (CNS). The emergence of human-associated SAg^+^ CNS would mark a prodigious shift in virulence capabilities. We examined CNS isolates from healthy human nares and diseased individuals, and determined that no known SAgs were present.

## Introduction

Staphylococcal superantigens (SAgs) are an important group of exotoxins. The SAg family consists of 23 members which includes toxic shock syndrome toxin-1 (TSST-1), the cause of nearly all cases of menstrual TSS and 50% of non-menstrual cases, staphylococcal enterotoxins (SEs), common causes of food poisoning and non-menstrual TSS, and SE-like (SE*l*) SAgs which lack or have not been tested for emetic activities, and whose roles in disease are under investigation [1]; SEs include SEA-SEE and SEG, and the SE*l* SAgs include SE*l*-H to X.

SAgs derive their name from their capacity to induce T-cell activation [2,3]. Classical antigens stimulate 0.01% of T-cells, while SAgs can stimulate 30% or more of T-cells [1–4]. SAgs recognize a specific subset of Vβ-chains of the T-cell receptor (Vβ-TCR) and crosslink the Vβ-TCRs and α and/or β-chains of major histocompatibility complex (MHC) class II molecules on antigen presenting cells (APCs) [1–3]. The crosslinking activates the T-cells and APCs leading to proliferation and massive release of cytokines [1–4].

Nearly all *S*. *aureus* strains produce one or more SAgs [1,5]. However, there are only a few reports that discuss SAg production by coagulase-negative staphylococci (CNS) [6–11]. In the early 1980s, a study concluded that human CNS did not produce the SAg TSST-1 [6]. However, more recent studies indicate that CNS isolated from veterinary sources and food may produce typical *S*. *aureus* SAgs [11,12]. These data led us to examine the presence or expression of SAgs in human CNS from multiple sources.

## Materials and Methods

### Ethics statement

The collection of staphylococcal isolates from 50 healthy human volunteer donors was performed under approved University of Minnesota IRB protocol 1103M97296. All volunteer donors gave written informed consent to participate. All other staphylococcal isolates that were evaluated in this study were submitted by physicians treating toxic shock syndrome patients. IRB approval to receive and test these specimens was not required since these were clinical samples submitted at the request of treating physicians and not part of a human clinical study.

### Staphylococcal isolates

Fifty healthy adult volunteers were analyzed for staphylococcal nasal colonization. Sterile cotton swabs were used to obtain individual samples from both nares of the volunteers. Staphylococcal isolates were identified by colony color on mannitol salt agar plates and morphology (gram-positive, cluster-forming cocci) and catalase activity. *S*. *aureus* and coagulase negative staphylococci were differentiated based on their coagulase activities (using rabbit plasma and slide coagulase test). Fourteen nasal *S*. *aureus* isolates were cultured from these healthy individuals. Additionally, 48 nasal CNS isolates were obtained from these same individuals. CNS typing of the 48 nasal organisms was performed using the bioMérieux api Staph identification system according to manufacturer’s instructions. Briefly, bacterial strains were plated on TSA blood agar plates and grown at 37°C overnight for 20 hrs. A homogenous bacterial suspension was prepared in API Staph medium to a turbidity of 0.5 McFarland and used to inoculate the api strip. Strips were read after incubation 37°C for 24 hrs. All 48 organisms were determined to be *S*. *epidermidis*.

Fifty-seven coagulase negative staphylococci from human patients with TSS as defined by the treating physician were submitted for SAg testing to the Schlievert laboratory between 1980 and 2007. We have previously shown that physicians have high accuracy in identifying TSS cases based on patient symptoms and signs of disease [13]. All of these submitted 57 CNS isolates were *S*. *epidermidis* as determined by the clinical microbiology laboratories of submitting medical centers. These isolates were tested for TSST-1, SEB, and SEC by antibody-based assays [14]. In multiple studies, we have shown that this antibody-based assay reliably detects production of these three SAgs [14–16]. This latter collection of isolates was not available for SAg gene testing by PCR as they were discarded after antibody testing with negative results for TSST-1, SEB, and SEC. However, 3 more recently submitted *S*. *epidermidis* isolates from TSS patients were available for SAg gene testing by PCR.

Collectively, three groups of isolates were compared for the presence of SAgs or their genes. Group 1 included the 14 *S*. *aureus* isolates from the nares of healthy individuals; these also served as controls for SAg detection. Group 2 included the 48 nasal *S*. *epidermidis* isolates from healthy individuals. Group 3 (clinical isolates) included 60 total *S*. *epidermidis* isolates recovered from the bloodstream of patients with TSS, and an additional 10 clinical *S*. *lugdunensis* isolates from the University of Iowa hospitals and clinics. Thus, we tested a total of 118 CNS for SAgs by antibody tests or their genes by PCR.

### Antibody and PCR assays for SAgs and their genes

CNS isolates from TSS patients (57) were tested by an antibody-based test for the three major SAg causes of TSS, notably TSST-1, SEB, and SEC. Briefly, the isolates were cultured at 37°C with shaking (200 rpm) overnight in 50 ml Todd Hewitt broth and then treated with 4 volumes of absolute ethanol to precipitate SAgs. Subsequently, the precipitates were resuspended in 0.1 ml of distilled water (500 times concentrated) and clarified by centrifugation (10,000 x g, 5 min). The clarified supernates were tested by double immunodiffusion for reaction with hyperimmune rabbit antisera specific for each toxin. The lower limit of detection of SAgs by this method was 0.01 ug/ml original culture fluid. In tests of thousands of *S*. *aureus* strains, TSST-1 is typically produced at 3.5 ug/ml, whereas SEB and SEC are produced at 25–100 ug/ml.

For all other isolates, including the three more recent CNS (*S*. *epidermidis*) isolates from TSS patients, the organisms were cultured in 10 mL of Todd Hewitt broth and then subjected to DNA extraction for use in PCR [17].The primers used and PCR amplification reactions were done as previously published [17].

## Results

### Nasal colonization

Of fifty healthy volunteers, 48 (96%) carried staphylococci in their anterior nares. Among the volunteers, 48 of 48 (100%) individuals were positive for CNS, and 14 out of 48 (29%) individuals were positive for both CNS and *S*. *aureus*. API strip tests determined that all nasal CNS isolates were *S*. *epidermidis* species. There was no individual who was colonized by coagulase-positive *S*. *aureus* in the absence of CNS.

### SAg gene profile of Staphylococcal isolates

**Fig 1:**
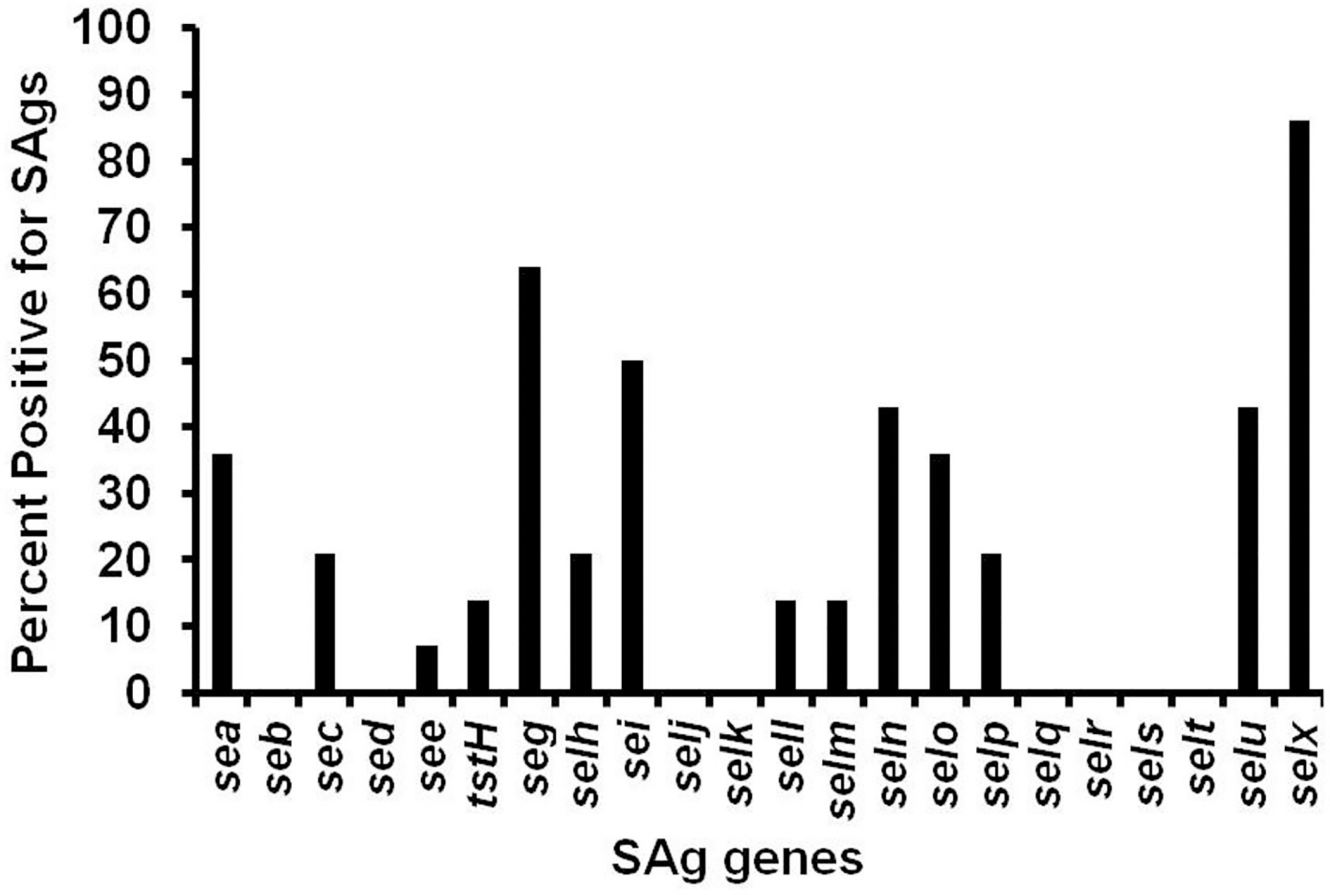
SAg gene profile of nasal *S*. *aureus* isolates. Conventional PCR and specific primers for known SAg genes were utilized [17].

Conventional PCR was used to screen for the presence of canonical SAg genes *sea-seg*, *tstH*, and *selh-selx* in three groups of staphylococcal isolates: (Group 1) nasal *S*. *aureus* isolates from 14 healthy individuals, (Group 2) nasal CNS isolates from healthy individuals (48 *S*. *epidermidis* isolates), and (Group 3) clinical CNS isolates (60 *S*. *epidermidis* and 10 *S*. *lugdunensis*) (Table 1). As expected, all *S*. *aureus* strains, in Group 1, carried genes encoding for SAgs with an average of 5 SAg genes per isolate (Table 2). Among them, *selx* was the most prevalent with 86% of distribution. *seg*, *seli*, *seln*, and *selu* also appeared in high frequency (greater than 40%). There was no detection of SEB, SED, SE*l*-J, SE*l*-K, SE*l*-Q, SE*l*-R, SE*l*-S, and SE*l*-T (Fig 1). Data for individual strain production of SAgs is shown in S1 Dataset. Unlike in *S*. *aureus* isolates, there was no known SAg gene detected among the 118 CNS (108 *S*. *epidermidis* and 10 *S*. *lugdunensis*) isolates in both Groups 2 and 3 regardless of their origin (Table 2). None of the 57 *S*. *epidermidis* isolates submitted for TSS-associated SAg testing to the Schlievert laboratory between 1980 and 2007 produced detectable TSST-1, SEB, or SEC, with lower limit of detection being 0.01 ug/ml. These isolates were not available for PCR testing in the current study. Three additional TSS *S*. *epidermidis* isolates from TSS patients were available for PCR analysis, and none of the organisms contained any of the known SAg genes; thus a total of 60 *S*. *epidermidis* isolates from TSS patients were negative for the three (TSST-1, SEB, SEC) SAgs known to cause most cases of TSS.

**Table 1:** Species and sources of the staphylococcal isolates.

Species (no. of isolates)	Sources (no. of isolates)
*S*. *aureus* (14)	Anterior nares^a^ (14)
*S*. *epidermidis* (48)	Anterior nares^a^ (48)
*S*. *epidermidis* (60)	TSS (60)
*S*. *lugdunensis* (10)	Endocarditis (3), Breast abscess (1), Finger Pulp (2), Others (4)

^a^ Samples obtained from nasal swabs of healthy volunteers.

**Table 2:** Prevalence of SAg genes in staphylococcal isolates.

Group	Total no. of isolates	No. of isolates producing known SAgs or having SAg genes	Average number of known SAgs or SAg genes per isolate
1 (*S*. *aureus*)	14	14	5 ± 2
2 (*S*. *epidermidis*)	48	0	0
3 (*S*. *epidermidis* and *S*. *lugdunensis*)	60 and 10	0 and 0	0 and 0

## Discussion

When TSST-1 was first described in 1981 [16,18], one of the original published manuscripts indicated that TSST-1 could be produced by CNS (*S*. *epidermidis*) [18]. A subsequent publication showed that at least some of those CNS that tested positive were in fact *S*. *aureus* strains, noting that TSST-1 positive *S*. *aureus* may give weak coagulase test reactions and appearing instead to be CNS [6]. However, the remaining CNS in that initial study were shown to be TSST-1 negative, with the original test results being false positives. Subsequent to the initial description of TSST-1, the Centers for Disease Control and Prevention (CDC) hypothesized that the TSST-1 gene originated in CNS and was transferred by bacteriophages to *S*. *aureus*. The Schlievert and Bergdoll (now deceased) laboratories both tested a sampling of *S*. *epidermidis* strains from unused tampons that had been isolated by the CDC to test their hypothesis. Both the Schlievert and Bergdoll laboratories showed that none of the *S*. *epidermidis* strains were positive for TSST-1 [6]. It was thus concluded that CNS strains do not produce TSST-1 and do not cause TSS in humans. It is noteworthy that despite these findings, CNS remain the number one cause of bacteremia in humans, many with symptoms that resemble TSS [19,20].

The original defining immunobiological activities of SAgs (formerly called pyrogenic toxins) are ability to cause fever and enhance host susceptibility to lethal endotoxin shock by up to 10^6^-fold in rabbits [21]. Subsequently, it was shown that these two activities depended on the ability of SAgs to stimulate T cells and antigen-presenting cells to produce massive amounts of cytokines; the origin of the term SAg to describe the toxins [3]. Because of the TSS-like symptoms observed in some patients with CNS infections, the Schlievert laboratory tested 22 *S*. *epidermidis*, 10 times concentrated culture fluids, for ability to induce fever and enhance endotoxin shock in rabbits; all 22 strains were negative, suggesting these CNS did not produce known or yet to be described SAgs that caused the TSS symptoms [6].

Recent studies indicate that known SAgs present in nearly all *S*. *aureus* can be occasionally present in CNS [7,10]. These results are in opposition with the above previous studies that established that CNS of human origin, primarily *S*. *epidermidis*, do not encode *S*. *aureus* SAgs [6]. In one study of a single *S*. *epidermidis* strain originally provided by the Bergdoll laboratory, the organism has been clearly established as both CNS and verified to produce *S*. *aureus* SAgs [7]. There have also been occasional other studies noting SAg production by CNS, particularly of animal or food origin [8,11]. Additionally, one study showed that 6.6% of 136 human CNS strains produced known SAgs (TSST-1 and/or SEA-SED) [10]. Interestingly, none of 35 *S*. *epidermidis* and none of 12 *S*. *lugenunensis* strains in that study produced known SAgs, in agreement with the findings from the 1980s [6] and our current study, wherein we showed that none of 118 *S*. *epidermidis* and *S*. *lugdunensis* strains produce any of the known SAgs. Thus, with one exception strain (FRI909) [7], the major CNS (*S*. *epidermidis* and *S*. *lugdunensis*) that infect humans do not produce known or unknown SAgs, despite their abilities to cause human diseases.

In a prior study, we tested a small number of CNS that only occasionally cause human infections, for example *S*. *hominis* and *S*. *capitis*, and showed none produced TSST-1, SEB, and/or SEC [6]. In contrast, in a recent study of human CNS, researchers in Japan showed that 10% of *S*. *hominis* and *capitis* strains from humans produce one or more of these same three SAgs [10]. These data differences make three important points: 1) CNS in Japan may be different from those isolated in the U. S., where to date no confirmed TSS cases have been associated with SAgs production by CNS; 2) even if CNS strains in Japan produce known SAgs, the SAgs do not cause TSS (none of the affected patients showed TSS symptoms); and 3) there are no data to suggest that CNS strains from humans in the U.S. should be routinely tested for SAgs as causative factors in TSS.

Our study indicates, as expected, that both *S*. *aureus* and CNS can be isolated from anterior nares of humans. While only 24% of tested individuals were positive for *S*. *aureus*, 96% were positive for *S*. *epidermidis* CNS. Interestingly, individuals colonized with *S*. *aureus* were also co-colonized with CNS, but when dually present, *S*. *aureus* dominated. This is in agreement with a previous study where we reported that *S*. *aureus* and CNS are inversely correlated in colonization of mucosal surfaces [22]. When we examined the CNS strains for the presence of SAgs, none of the isolates encoded for SAg genes, whereas *S*. *aureus* isolates encoded an average of 5 SAgs per strain. To date, this is the only study to evaluate a large collection of *S*. *epidermidis* strains for the presence of any of the 23 known staphylococcal SAg genes.

## Supporting Information

S1 Dataset The SAg profile of all strains used in this study are provided (S1 Dataset). These include the 14 *S*. *aureus* strains and 51 *S*. *epidermidis* strains tested by PCR, 10 *S*. *lugdunensis* strains tested by PCR and 57 S. epidermidis isolates from TSS patients tested by antibody reaction against hyperimmune serum raised specifically against TSST-1, SEB, or SEC.
(XLS)
